# Do Rurality‐Based Financial Incentives Improve Equity of Primary Healthcare Access? Evidence From Australia

**DOI:** 10.1002/hec.70000

**Published:** 2025-06-18

**Authors:** Karinna Saxby, Yuting Zhang

**Affiliations:** ^1^ The Melbourne Institute of Applied Economic and Social Research The University of Melbourne Melbourne Australia

**Keywords:** Australia, financial incentives, inequity, policy evaluation, primary care, rural

## Abstract

In Australia, as in many other countries, people living in rural and remote areas experience poorer health outcomes and use less primary healthcare compared to urban populations. Aiming to reduce these inequities, in 2022 the Australian government increased rural‐based financial incentives for General Practitioners (GPs) to “bulk bill” (i.e., provide care with zero patient out‐of‐pocket costs) children and concession card holders (low‐income patients and older adults) living in rural and remote, but not urban areas. Using whole‐of‐population administrative data and exploiting variation in the eligibility of geographic areas to receive these incentives, we find that, compared to people living in urban areas, the reform led to a 2.7% (95% CI 2.2; 3.2) increase in the number of GP visits, a 9.0% (95% CI 8.4; 9.5) increase in the number of bulk billed GP visits, and a 13.0% (95% CI 12.4; 13.7) reduction in the out‐of‐pocket cost per GP visit among people living in rural areas. Effects were more pronounced for people with higher initial out‐of‐pocket costs—adults rather than children, people without concession cards, and people living in areas with less socioeconomic disadvantage. Altogether, while the reform has gone some way to reduce out‐of‐pocket costs for rural patients, benefits are unequal and inequities in access remain.

## 1 Introduction

Equitable and affordable access to healthcare, especially primary care, is a key objective of many countries with universal healthcare (Goddard and Smith 2001; Van Doorslaer et al. 2008; Frenz and Vega 2010). Yet, inequities in access are persistent across different population groups and geographies. Compared with urban populations, rural populations experience poorer health outcomes, have less access to, and use of, primary and preventive healthcare, and, partially due to a lack of primary care access, experience higher hospitalization rates (Casey et al. 2001; Pong et al. 2009; Wilson et al. 2009; Douthit et al. 2015; Hirello et al. 2022).

The costs of healthcare for patients in rural and remote areas is a well‐established barrier to accessing care (Palmer et al. 2013; Douthit et al. 2015). Notwithstanding personal costs associated with traveling larger distances to seek care, rural populations are more likely to incur higher out‐of‐pocket healthcare costs compared to those in metropolitan regions (Gravelle et al. 2016; Saxby and Zhang 2024).

Understanding the causal effects of interventions in this space remains vital given persistent health inequalities experienced by rural populations and unique barriers pertaining to accessibility and cost of healthcare. With approximately 23% of the population (4.8 million people) living in rural areas and 2% (∼331,000 people) living in remote and very remote communities (ABS 2024), the Australian context provides a unique opportunity to explore whether targeted interventions can reduce rural‐urban disparities in access to primary care.

Here we explore the impact of an Australian policy that introduced financial incentives for GPs to “bulk bill” (i.e., provide care with zero patient out‐of‐pocket costs) children and concession card holders (low‐income patients and older adults) living in rural and remote, but not in urban areas (Department of Health and Aged Care 2023a). These incentives were also tiered by rurality; with larger incentives provided to GPs practicing in remote and very remote areas compared to those in rural areas. Specific policy objectives of the reform were to (1) increase financial viability and supply of GPs practicing in rural and remote areas and (2) increase access to health services and make healthcare more affordable for people living in these regions.

Using linked whole‐of‐population administrative data on GP visits, bulk billed GP visits, and out‐of‐pocket costs between 2021 and 2022, here we evaluate whether the reform was, by its own definition, a success. We exploit the framework of a natural experiment, whereby we compare outcomes for patients living rural areas before and after the reform to individuals living in urban (i.e., untreated) regions over the same period. To explore the short‐term impacts on supply in rural and remote areas, we also conduct descriptive analyses using national health workforce data. Specifically, we investigate whether there were changes in the number of GPs practicing across different rurality classifications, or in their working hours, between 2021 and 2023.

We find that the reform led to a 2.7% (95% CI 2.2; 3.2) increase in the number of GP visits, a 9.0% (95% CI 8.4; 9.5) increase in the number of bulk billed (free) GP visits for people living in rural areas relative to people living in urban areas – closing the pre‐reform rural‐urban gaps by approximately 34% and 77%, respectively. The reform also led to a 13.0% (95% CI 12.4; 13.7) reduction in the out‐of‐pocket cost per GP visit for rural patients. The reduction in out‐of‐pocket costs were larger for non‐targeted patients who initially had higher out‐of‐pocket costs, namely general rather than concessional patients and adults rather than children. Effects were also unevenly distributed by area‐level factors. While absolute (but not relative) effects were more pronounced in areas where the incentives were larger, reductions in out‐of‐pocket costs were concentrated to areas with higher socioeconomic resources. Descriptive evidence suggests that the reform has not led to an increase in GP availability or working hours in rural and remote areas. Altogether, while the reform improved affordability of GP visits for rural patients, the uneven effects and workforce analyses suggest that supply factors will continue to constrain the policy's overall effectiveness.

These results align with the growing literature on the effects of financial incentives and cost‐sharing in healthcare (Card et al. 2008; Scott et al. 2008; Carrieri 2010) (Winkelmann 2004) and specific literature demonstrating that targeted initiatives can play a role in closing disparities in primary care access for specific populations, albeit with uneven effects (Saxby et al. 2023). This research also links to the stream of international literature on how the expansion of social healthcare, or more broadly greater reimbursement, in rural areas can increase healthcare utilization and achieve better financial protection for patients, including in China (Wagstaff et al. 2009; Huang and Wu 2020), India (Aiyar and Sunder 2024; Singh 2024), and Canada (Laurent 2002).

This paper makes several distinct contributions. While several papers have looked at the impacts of the earlier iterations of rural incentives on GP supply and waiting times in Australia (Yong et al. 2018; Swami and Scott 2021), to our knowledge, we are the first to investigate the whole‐of‐population, patient‐level effects of these tiered rurality incentives on access and affordability of primary care services for patients in rural areas.

We also show how the policy effects varied across different patient groups and geographic areas and provide evidence on the uneven distribution of the benefits from the policy, with larger reductions in out‐of‐pocket costs observed for non‐targeted patients who initially had higher out‐of‐pocket costs. This echoes previous findings that GPs can respond differently in response to these incentives (Wong et al. 2017) and benefit patients with more socioeconomic resources (Saxby et al. 2023; Zhang and Chen 2024).

Given these distributional effects, more research is needed to understand the ultimate welfare implications of this policy. For example, it will be important to understand how the magnitude of this out‐of‐pocket cost reduction compares to the government contributions and whether the policy could have positive impacts on patient outcomes, such as reduced hospitalizations or improvements in health and wellbeing. Future research should therefore investigate the effects of these reforms on the revenue for GPs practicing in rural areas and ascertain whether closing these disparities in access can improve patient health.

These findings have important policy implications. Other countries facing similar inequities in healthcare access—particularly in rural or disadvantaged areas—could consider implementing place‐based incentives for healthcare providers. The specific administrative approach is likely to vary by context. For instance, in countries like China and India, rurality is measured using composite indices that consider a combination of population density and socioeconomic indicators (e.g., poverty status, per capita income, economic activity) (Wang et al. 2014; Singh 2024). While the administrative application may differ, place‐based affirmative action may yet be a useful tool to address geographic disparities in healthcare use and health.

The remainder of this paper is set out as follows. Section 2 provides an overview of the institutional background of Australia's universal health insurance programme and the policy reform. Section 3 and 4 respectively describe the data and empirical strategy. Section 5 presents the results of the analyses. Section 6 concludes by summarizing the key findings and discussing the policy implications.

## 2 Institutional Setting and Policy Change

All Australian citizens and permanent residents are entitled to free or subsidized healthcare under Australia's universal health insurance scheme, Medicare. Medicare provides free services in public hospitals and subsidizes out‐of‐hospital medical services and prescription medicines to varying degrees (Krassnitzer 2020). The subsidized services, which comprises more than 10,000 items, are listed on the Medicare Benefits Schedule (MBS).

Medical practitioners can set their own fees for services listed on the MBS and these fees are not regulated. If the provider charges the same fee as the benefit paid by the government (i.e., equal to 100%, 85% or 75% of the set schedule fee pending the service type), this service is said to be “bulk billed” and the patient incurs no out‐of‐pocket cost (Department of Health 2020). If service providers charge a fee above the benefit paid (or indeed, charges the MBS schedule fee or higher for non‐GP services) the individual pays the gap between the schedule fee and the benefit paid.

On top of the benefit paid, GPs are provided with additional financial incentives to bulk bill concession card holders and children under 16 (Van Der Weyden 2004; Kecmanovic and Hall 2015). Moreover, since 2004, GPs practising in rural areas have been provided with bonus payments via Rural Bulk Billing Incentive. These rural incentives are based on a specific index known as the Monash Modified Model (MMM). The MMM is based on population size and distance to urban centers and classifies rurality level into seven categories: MMM1: Metropolitan areas; MMM2: Regional centers; MMM3: Large rural towns; MMM4: Medium rural towns; MMM5: Small rural towns; MMM6: Remote communities; and MMM7: Very remote communities. Before January 2022, the bulk billing incentives were $AU6.90 in metropolitan (MMM1) areas and $AU10.45 in other areas (MMM2‐7).

**FIGURE 1:**
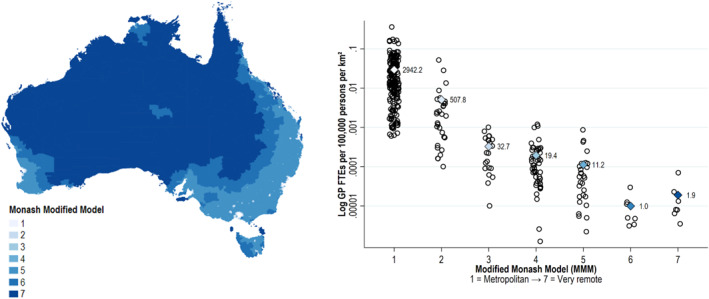
Geographical distribution of Monash Modified Model (MMM) classifications (left) and supply of GPs across MMM regions in 2021 (right). GP = general practitioner; MMM = monash modified model; SA3 = Statistical Area 3. The MMM classifies metropolitan, regional, rural and remote areas in Australia into seven categories: MMM1 = metropolitan areas; MMM2 = regional centers; MMM3 = large rural towns; MMM4 = medium rural towns; MMM5: small rural towns; MMM6 = remote communities; and MMM7 = very remote communities. Left panel shows geographical distribution of MMM regions. Right panel shows GP availability by MMM classification, with each dot representing a SA3 region and diamonds showing MMM group means (raw units). GP availability based on SA3‐level data in 2021 from Australia's national health workforce data tool (Department of Health and Aged Care 2023c). Full time equivalent (FTE) GPs per SA3 presented per population size and area to reflect not only differences in supply, but also the geographic availability of care—that is, in rural and remote areas, SA3 regions cover vast regions.

The variation of the MMM across different regions in Australia and the corresponding supply of GPs per MMM group is presented in Figure 1. It highlights the maldistribution of GP availability by remoteness: in MMM1 areas, there are approximately 2942 full‐time‐equivalent (FTE) GPs per 100,000 persons per km^2^. This drops sharply to just 1–2 FTE GPs per 100,000 persons per km^2^ in MMM6 and MMM7 (remote and very remote) regions.

Limited access to GPs in rural and remote areas has significant implications for affordability and use of primary care. In metropolitan areas, patients have more providers to choose from, including those who bulk bill. GPs in urban regions also have more competitors and thus incentives to charge lower prices and reduce patient out‐of‐pocket costs (Gravelle et al. 2016). Conversely, patients in rural and remote areas often face limited provider choice, incur higher out‐of‐pocket costs, and need to travel longer distances to access care (Linnane et al. 2024; Saxby and Zhang 2024). These inequities in access to primary healthcare in rural areas has been associated with reduced utilization of primary care, poorer health outcomes, including suboptimal management of chronic diseases, and higher rates of potentially preventable diseases and avoidable hospitalizations (Casey et al. 2001; Pong et al. 2009; Thomas et al. 2015; Hirello et al. 2022; Yisma et al. 2025).

These persistent inequities in access and affordability are a well‐recognized policy issue in Australia. Recent studies highlighting that affordability remains a key barrier to healthcare use in rural areas (Linnane et al. 2024; Saxby and Zhang 2024) and that, compared to people living in urban regions, rural Australians are more likely to forgo or delay using healthcare due to cost (ABS 2015).

With the aim to improve affordability and access to primary care for rural patients, and increase financial viability of rural practices, the Australian government recently introduced changes to the Rural Bulk Billing Initiative (Department of Health and Aged Care 2022; RACGP 2022). Specifically, in January 2022, the government increased bulk billing incentives for rural and remote areas from $AU10.45 to: $AU11.05 in MMM3‐4 regions, $AU11.75 in MMM5 regions, $AU12.40 in MMM6 regions, and $AU13.15 in MMM7 regions. In November 2023, they expanded this benefit even further by tripling the bulk billing incentive payments for concessional patients and children (Department of Health and Aged Care 2023b). In this paper, we investigate the first stage of this incentive.

## 3 Data

The data for this analysis comes from the Person‐Level Integrated Data Asset (PLIDA), an individual‐level linked dataset which combines information from population Census and various administrative data sources including healthcare use, social security records, income and taxation, and employment (Saxby et al. 2020; ABS 2022).

For this study, we source data from the 2021 Census and the 2021‐2023 Medicare records. For computational efficiency, we retain a 10% random sample of Australians that completed the 2021 Census. Using individuals' residential location at the time of the Census, we then link in data on weighted average MMM rurality classifications at the Statistical Area 3 (SA3) level.^1^ Following previous research, MMM1 and MMM2 areas were classified as “urban,” MMM3‐MMM7 areas were classified as “rural” (Saxby and Zhang 2024).

One limitation with the current data is that we do not observe individuals who move MMM classifications during this time. To estimate the extent to which this may impact our estimates, we explore how many individuals move SA3 regions and MMM classifications in an external population representative longitudinal dataset, the longitudinal Household Income and Labor Dynamics in Australia (HILDA) survey (Summerfield et al. 2022). We find that around 9% of people move SA3 regions each year, and only 3% move MMM regions.^2^ This suggests that most people move to similar rurality classifications and that migration is expected to have a relatively small impact on our estimates.

Information on GP visits, bulk billed GP visits, and associated patient costs are provided through Medicare claims^3^ and aggregated to the individual‐quarter level from quarter 12,021 to quarter 32,023.

Descriptive characteristics by rurality are presented in Table 1. Compared to individuals living in urban regions, individuals living in rural regions were slightly older (mean 41 vs. 39 years), were more likely to report a core activity limitation (0.07 vs. 0.06) or long‐term health condition (0.37 vs. 0.35), had lower levels of educational attainment, and were more likely to be a concession card holder (0.43 vs. 0.36). Urban regions were more densely populated than rural regions and had lower levels of socioeconomic disadvantage (27% of individuals living in urban regions lived in the areas with higher disadvantage, compared to 93% in rural areas). Individuals living in rural areas used fewer GP services than those in urban areas (0.94 vs. 1.00 GP visits per quarter) and had fewer bulk billed GP services (0.70 vs. 0.74) but incurred slightly higher out‐of‐pocket costs per GP visit each quarter ($15.2 vs. $13.9).

**TABLE 1:** Descriptive characteristics of study sample in 2021.

	Urban (*n* = 726,720)	Rural (*n* = 174,022)
	Mean/prop.	Mean/prop.
Individual‐level characteristics		
Age	39.4	41.3
Female	0.51	0.50
Concession card holder	0.36	0.43
Core activity limitation	0.06	0.07
Long‐term health condition	0.35	0.37
Quartile equivalised household income		
1 (lowest quartile)	0.28	0.35
4 (highest quartile)	0.22	0.15
Educational attainment		
Less high school	0.13	0.19
Uni or above	0.21	0.11
Region‐level characteristics		
SA3 population	95,156	52,729
Rurality (MMM index)		
1 (metropolitan)	0.81	—
2	0.19	—
3	—	0.25
4	—	0.36
5	—	0.27
6	—	0.06
7 (remote)	—	0.05
Area‐level disadvantage		
Lower area‐level disadvantage	0.49	0.01
Higher area‐level disadvantage	0.27	0.93
Outcomes (per patient‐quarter)		
Number of GP visits	1.00	0.94
Number of bulk billed GP visits	0.74	0.70
Out of pocket cost toward GP visits ($)	11.00	10.97
Out of pocket cost per GP visit ($)	13.85	15.20
Out of pocket cost per non bulk billed GP visit ($)	42.31	45.71

*Note:* Urban regions are MMM1‐MMM2 regions, rural regions are MMM3‐MMM5 regions. Individual‐ and regional‐level characteristics are based on values reported at the time of the 2021 Census. Outcomes are based on all quarters of data in study sample period, that is, quarter one 2021 to quarter three 2023 Medicare data. Lower/higher area‐level disadvantage based on top/bottom two quartiles of area‐level disadvantage as per the Index Regional Socioeconomic Disadvantage (IRSD) score.

Abbreviations: GP = general practitioner; MMM = Monash Modified Model; SA3 = Statistical Area 3.

## 4 Empirical Strategy

To estimate the policy effects, we compare outcomes among individuals living in rural regions pre and post reform with outcomes among individuals living in urban regions over the same period. In this framework, we refer to individuals living in rural areas as the “treated” group as they were affected by the policy whereas individuals living in metropolitan regions serve as the “non‐treated” or “control” group as they were not affected by the policy.

This difference‐in‐differences approach assumes that the differences in outcomes between individuals living in rural and remote areas relative to individuals living in urban regions would have continued to evolve in parallel had the reform not been introduced. Because this assumption is crucial for identification, it is necessary to impose several restrictions to our sample.

First, we look at outcomes between quarter one 2021 and quarter three 2023—that is, four quarters before and seven quarters after the reform. This pre‐reform time frame is important as during the COVID pandemic there were lockdowns which impacted individuals' access to healthcare. These lockdowns were also largely patterned by rurality. For example, during 2020, metropolitan regions were disproportionately impacted by lockdowns; and GP consultations reduced (Siette et al. 2021; Savira et al. 2023). Similar patterns were also observed during 2021 with additional lockdowns occurring in metropolitan regions of Victoria, New South Wales, and the Australian Capital Territory. To address this, we exclude the 2020 period and exclude these regions where there were these additional lockdowns in 2021. Last, we only look at the first seven quarters after the reform (up to quarter four 2023). This is because, as aforementioned, the Australian government introduced additional reforms to Medicare in November 2023. By narrowing the time frame, we additionally aim to isolate the more immediate impacts of the reform.

**FIGURE 2:**
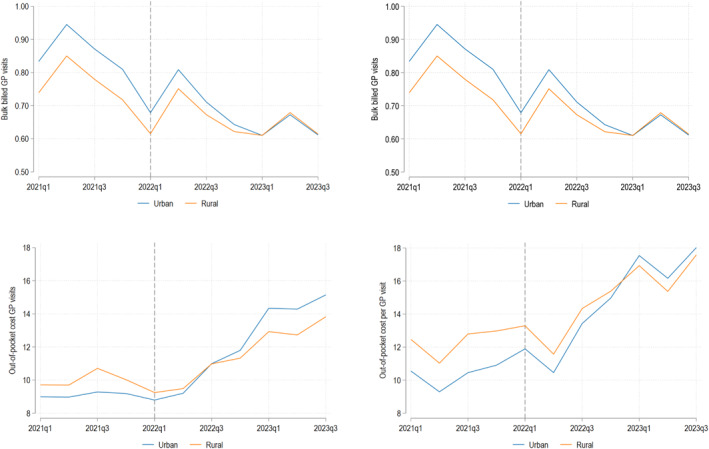
Temporal trends of outcomes by urban and rural areas. Mean unadjusted quarterly outcomes for estimation sample from quarter one 2021 to quarter three 2023.

The temporal trends in outcomes for our estimation sample are presented in Figure 2. These figures show the unadjusted average number of GP visits, number of bulk billed GP visits, the out‐of‐pocket cost toward GP visits, and the out‐of‐pocket cost per GP visit per patient‐quarter in urban and rural regions between quarter one 2021 and quarter three 2023. More broadly, these descriptive results provide important policy context, indicating that, during this time, the number of bulk billed GP visits had been decreasing and the out‐of‐pocket costs toward GP visits had been increasing.

Consistent with our descriptive statistics, prior to the reform, the number of GP visits and bulk billed GP visits in rural areas were lower than those in urban areas but the out‐of‐pocket costs were higher. These temporal trends also indicate that pre reform, the differences in outcomes generally appear to evolve in parallel. We confirm this by formally testing for pre reform differences in trends (Supporting Information S1: Appendix A.1).^4^ Post 2022, these figures provide some descriptive evidence that the policy may have reduced rural‐urban differences in outcomes; the number of bulk billed GP visits increased in rural areas while the out‐of‐pocket cost toward GP visits reduced.

To empirically estimate the effects of the policy, we use ordinary least squares (OLS) regression to look at the total number of GP visits, the number of bulk billed GP visits, the out‐of‐pocket cost towards GP visits, and, to isolate the price effect, the out‐of‐pocket cost per GP visit, per patient‐quarter in the following form:

(1) $$y_{irt}=\alpha_{i}+\beta_{1}D_{ir}+\beta_{2}\left(D_{ir}\times T_{t}\right)+A_{it}+X_{i}+\rho_{t}+\varepsilon_{it}$$

Where y is the outcome of interest for individual i living in SA3‐region r in quarter t, $D_{ir}$ is an indicator variable equal to 1 if the individual resided in a treated region (i.e., a rural or remote region), $T_{t}$ is equal to “0” before the implementation of the policy and “1” after quarter one 2022. In $\rho_{t}$ we control for quarter‐year fixed effects. In $A_{it}$ we control for individuals' age in bins (5–14, 15–24, 25–34, …, 75–84, 85 years and above) and in $X_{i}$ we control for sex (“male,” “female”) based on the 2021 Census response. The main coefficient of interest from Equation 1, $\beta_{2}$, represents how outcomes change among individuals living in treated regions post policy change. Standard errors are clustered at the individual level.

Following the main estimation, we conduct several robustness checks and alternate specifications.

First, we consider the robustness of our results to alternate functional forms. In particular, we apply alternate models to account for the highly skewed and zero‐inflated distributions our outcome variables (Supporting Information S1: Appendix A.2^5^). We estimate a Poisson model, a negative binomial model, and a zero‐inflated Poisson model for count outcomes (GP visits, bulk billed GP visits). For out‐of‐pocket costs, we additionally estimate a linear probability model on the probability of incurring positive out‐of‐pocket costs and then a Poisson model, conditional on having positive out‐of‐pocket costs.

We then conduct numerous heterogeneity analyses. First, we expect that as the rural‐based incentives are only applied for children under the age of 16 years and concessional patients, that the effects may be larger for these groups. However, as the reforms are targeted to population groups that already receive higher rates of bulk billing (i.e., children and concession card holders) (Elkins and Schurer 2017), the additional revenue received by GPs who already bulk bill these patients could have spillover effects to other population groups living in “treated” areas. To investigate these potential effects, we estimate the models separately by age group (Less 16 years, 16–39 years, 40–64 years, 65 years and above), and patient category (concession card holders, general). To further investigate “intensity” of treatment, we then explore whether the reform had larger effects in areas where the incentives to bulk bill were higher. Specifically, we look at the heterogeneous effects for MMM3‐4 regions (where the incentive increased by 5.7%), MMM5 regions (where the incentive increased by 12.4%), and MMM6 and MMM7 regions (where the incentive increased from by 18.7% and 25.8% respectively).

We additionally test whether effects vary based on the socioeconomic resources of the area (above/below median area‐level disadvantage).^6^ As areas with higher levels of socioeconomic disadvantage have a higher share of concession card holders (Wong et al. 2017), it is possible that the higher GP revenue associated with bulk billing these patients may lead to larger reform effects in these regions. However, as there is greater supply of healthcare providers in areas with relatively higher area‐level resources, and in turn greater price competition (Gravelle et al. 2016), it is possible that we may see larger reform effects in areas with greater resources. For simplicity, we restrict the heterogeneity analyses to GP visits, the number of bulk billed GP visits, and the out‐of‐pocket cost toward GP visits.

Finally, to provide descriptive evidence on how the policy may have impacted supply or availability of GPs, we source data from an external dataset: the National Health Workforce Dataset (Department of Health and Aged Care 2023c). This dataset provides information on the number of Full Time Equivalent (FTE) GPs, and their average working hours (in categories) across different SA3 regions in Australia over time. To investigate whether there were short‐run impacts on GP supply by rurality, we once again assign MMM categories to each SA3 region and calculate the number of FTE GPs, and their average working hours, by rurality over 2021–2023.

## 5 Results

### 5.1 Main Results

The main results estimated by OLS are presented in Table 2. These show the adjusted pre reform differences in outcomes for individuals living in rural regions compared to urban regions (β1) and how these differences changed post policy (β2). Prior to the reform, individuals living in rural areas used slightly fewer GP services [−0.11 (95% CI −0.12; −0.11) per quarter], received fewer bulk billed GP services [−0.13 (95% CI −0.13 0.12) per quarter] and incurred higher out‐of‐pocket for GP services [2.22 (95% CI 2.12; 2.33) more per visit] relative to those living in urban regions.

**TABLE 2:** Results from difference‐in‐differences regression analyses.

	Number of GP visits		Number of bulk billed GP visits		Out‐of‐pocket cost toward GP visits		Out‐of‐pocket cost per GP visit	
	β [95% CI]	Relative change (%)	β [95% CI]	Relative change (%)	β [95% CI]	Relative change (%)	β [95% CI]	Relative change (%)
Rural ($\beta_{1}$)	−0.111*** [−0.117; −0.105]	—	−0.126*** [−0.132; −0.120]	—	0.841*** [0.737; 0.945]	—	2.222*** [2.115; 2.329]	—
Post × rural ($\beta_{2}$)	0.027*** [0.022; 0.032]	2.7 [2.2; 3.2]	0.069*** [0.065; 0.073]	9.0 [8.4; 9.5]	−1.505*** [−1.599;−1.411]	−15.0 [−15.9; −14.1]	−1.751*** [−1.845;−1.658]	−13.1 [−13.8; −12.4]
**No. Observations**	**9,908,162**		**9,908,162**		**9,908,162**		**4,797,928**	
Unadjusted sample means (pre reform)								
Urban	1.07		0.86		9.11		11.06	
Rural	0.99		0.77		10.04		13.42	
Difference (rural‐urban)	−0.08		−0.09		+0.93		+2.37	

*Note:* Percentage change is relative to unadjusted sample mean among individuals living in rural areas in all quarters before the reform (provided in table). Unit of observation is at patient‐quarter level. Models control for age, sex (“male,” “female”), and quarter‐year fixed effects. Rurality is based on the Monash Modified Model of remoteness with MMM1‐MMM2 being classified as urban regions and MMM3‐MMM7 being classified as rural regions. Standard errors are clustered at the individual level.

Abbreviation: 95% CI = 95% confidence intervals.

*p < 0.05.

**p < 0.01.

*** *p* < 0.001.

After the additional rural incentives were introduced, the number of GP visits and bulk billed GP visits increased among individuals living in rural areas [0.03 (95% CI 0.02; 0.03) and 0.07 (95% CI 0.07; 0.07) per patient‐quarter, respectively], relative to individuals living in urban areas. This represents an approximate 2.7% increase in GP visits and a 9.0% increase in bulk billed GP visits for patients in rural areas relative to their baseline levels; closing the rural‐urban gap in GP visits and bulk billed GP visits by 34% and 77%, respectively. The average out‐of‐pocket cost per GP visit also fell by $1.75 (95% CI 1.66; 1.85)—approximately 13.0%—for individuals living in rural areas. Results were largely unchanged when estimated using Poisson, zero‐inflated Poisson, or negative binomial models for GP visits (estimates ranging from 1.8% to 2.5% more GP visits in rural areas post reform) and bulk billed GP visits (estimates ranging from 4.5% to 9.3% more bulk billed GP visits in rural areas post reform) (Supporting Information S1: Appendix A.3). The two‐part model estimated on the out‐of‐pocket costs toward GP visits indicates that the reduction in out‐of‐pocket costs is driven by the extensive margin—that is, the policy reduces the likelihood that individuals will incur any out‐of‐pocket cost [by 2.7% (95% CI 2.6; 2.8)]. This aligns with the policy design, which targets the extensive margin by influencing GPs' decision to bulk bill. Indeed, among individuals in rural areas who are not bulk billed post reform, out‐of‐pocket costs actually increase [by 2.7% (95% CI 2.1; 3.3)].

### 5.2 Heterogeneity Analyses

**FIGURE 3:**
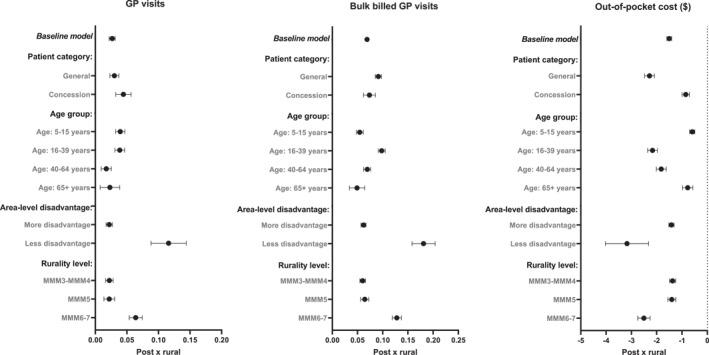
Heterogeneity analyses. Baseline models indicate results from all study samples as shown in Table 2. All models control for age, sex, and quarter‐year fixed effects. Standard errors are clustered at the individual level. Rurality level based on Monash Modified Model (MMM), with the following classifications MMM1 = metropolitan areas; MMM2 = regional centers; MMM3 = large rural towns; MMM4: medium rural towns; MMM5 = small rural towns; MMM6 = remote communities; and MMM7 = very remote communities.

The heterogeneity analyses showing how the reform impacted outcomes across different age groups, concession card category, area‐level resources, and rurality (“treatment intensity”) level are presented in Figure 3. Full results of the heterogeneity analyses for all outcomes are available in Supporting Information S1: Appendix A.4. Consistent with the baseline results, we find that across all subgroups investigated, people living in rural areas visit the GP less often and receive fewer bulk billed GP services than those in urban areas. Of note, the rural‐urban differences in GP visits pre‐reform was larger among children and concession card holders.

Post reform, we observe that there were larger increases in GP visits and more pronounced reductions in out‐of‐pocket costs for patients who initially had higher out‐of‐pocket costs—general patients rather than concessional patients and adults rather than children. Given the incentives are explicitly tied to concessional patients and children, this could suggest that the additional revenue from GPs bulk billing these “targeted” patients may have offset the fees charged to, or propensity to bulk bill, other patients in rural areas. As rural‐urban differences in out‐of‐pocket costs for children and concession card holders were initially smaller pre‐reform, it follows that the policy impacts on the demand for GP visits among these targeted populations may be less pronounced. This points to potential spillover benefits of the policy for some rural patient groups, but highlights the persistence of gaps for others.

We also find there are heterogeneous effects across area‐level characteristics. Absolute effects were larger in areas where incentives were higher, particularly in remote and very remote regions (MMM6‐MMM7). However, when interpreting these coefficients relative to the pre‐reform differences (Supporting Information S1: Appendix A.6), we find that the policy had larger effects in MMM3‐MMM4 areas (reducing the gap by 29%) than MMM5 and MMM6‐MMM7 areas (reducing the gap by 20% and 22% respectively). This suggests that while incentives did have an effect in more remote areas, supply‐side constraints (such as workforce availability and service capacity) in very remote locations may have limited the overall impact of the policy relative to the existing disparities.

Lastly, we observe that absolute effects were less pronounced among patients living in areas with less socioeconomic resources. Pre‐reform rural‐urban differences show that patients in these areas were already more likely to be bulk billed and had lower out‐of‐pocket costs (Supporting Information S1: Appendix A.4), which may have limited the potential for further fee reductions. This again reinforces that there were larger reductions in out‐of‐pocket costs for patients who initially had higher out‐of‐pocket costs.

### 5.3 GP Availability

The number of FTE GPs per capita‐km^2^ and the average number of working hours for GPs across rurality is presented in Supporting Information S1: Appendix A.5 and Appendix A.6 respectively. We find that compared to 2021 figures, by 2023 (post reform), the number of FTE GPs per population had increased in metropolitan areas but decreased in rural areas. Compared to 2021 figures, working hours in 2023 had reduced across all regions, but this was more pronounced in rural and remote areas. Although this is a largely descriptive analysis, this could suggest that the policy did not cause substantial shifts in terms of GPs sorting out of metropolitan areas, at least in the short‐term.

## 6 Discussion

In this paper, we have evaluated the effects rural bulk billing incentives on GP visits and out‐of‐pocket costs in Australia. We found that the reform increased the utilization of GP visits in rural areas, but did not close the rural‐urban gap in the number of GP visits. The reform also increased the number of bulk billed GP visits (free GP visits) and reduced patient out‐of‐pocket costs in rural areas, relative to urban areas, which to a large degree closed the gaps in out‐of‐pocket costs between rural and urban areas. Effects were more pronounced in areas where the incentives were higher and in areas with lower levels of socioeconomic disadvantage.

These results align with the previous literature on the link between expansion of social healthcare, or more broadly greater reimbursement, in rural areas and increased healthcare utilization and better financial protection for patients (Wagstaff et al. 2009; Huang and Wu 2020; Aiyar and Sunder 2024; Singh 2024). These results are also consistent with the large literature on reduced patient cost sharing and increases in the demand for healthcare (Winkelmann 2004; Atella et al. 2006; Finkelstein 2007). The heterogeneous effects also align with research in Australia which has shown that changes to financial incentives and patient co‐payments had larger impacts on patients with initially higher out‐of‐pocket costs and among those living in regions with better supply (Wong et al. 2017; Saxby et al. 2023). Lastly, our descriptive analysis on GP availability also echoes previous research indicating that financial incentives have limited impacts on GP supply in rural and remote Australia (Broadway et al. 2017; Swami and Scott 2021).

These results should be considered within the context of several limitations. First, due to the introduction of the triple bulk billing incentive only seven quarters after the policy, it is difficult to explore the long‐term impacts of this policy. Other studies indicate that better financial protection for patients can lead to improved health outcomes and reduced acute healthcare costs, such as fewer hospital admissions (Huang and Wu 2020; Aiyar and Sunder 2024). As the PLIDA data does not currently contain information on acute healthcare utilization or other health measures, we cannot observe whether this policy impacted health outcomes or downstream costs such as Emergency Department visits and hospitalizations. However, it is important to note that there are ongoing efforts to link this information into PLIDA. Should this data become available, this will be a crucial avenue for future research. It is also important to note that with administrative data such as PLIDA, there are important data lags that may influence our estimates, such as updates to individuals' locations over time. This is particularly relevant when undertaking evaluation of relatively recent policies. Continued efforts to improve the timeliness of access to administrative data are encouraging and should be supported.

Finally, with current data gaps on the healthcare provided through Aboriginal Community Controlled Health Organizations (ACCHOs) (Saxby and Stephens 2024), it is also difficult to understand the specific policy effects among Indigenous populations in remote communities. However, it is likely that these policies are insufficient to address inequalities without other important enablers to access, such as models of inclusive and culturally appropriate care (Saxby and Stephens 2024). For example, other studies have shown that targeted price reductions and practice incentives have been insufficient to close the disparities in use of GP visits and prescription medications for Indigenous populations in rural and remote areas (Saxby et al. 2023). The specific policy effects on Indigenous populations should be considered in future research and in collaboration with ACCHOs and health consumers.

These results have several important policy implications.

First, consistent with the aims of the policy, it appears that rurality‐based incentives were effective in increasing GP visits and reducing the out‐of‐pocket costs towards primary care for rural patients, however these effects were uneven and insufficient to completely reduce disparities in access experienced by rural patients relative to urban patients. The heterogeneous policy effects also suggest there is scope for refining the incentive design to be both broader and more effective. For example, although pre‐reform rural‐urban disparities in GP visits were larger for children and concession card holders, their relatively higher bulk billing rates may have reduced scope for further fee reductions. This points to the need for complementary strategies to address persistent access barriers and promote more equitable access to primary healthcare services for rural patients.

Further, we found that, although the pre‐reform disparities in access and out‐of‐pocket costs increased with area‐level remoteness, the relative (but not absolute) impact of the policy in these areas was less pronounced. This could suggest that the efficacy of these incentives is constrained by supply in more remote parts of Australia. These results underscore the importance of considering both area‐level resources and individual‐level socioeconomic disadvantage when designing financial incentives. Policymakers might need to adjust the incentive structure to explicitly account for existing disparities, ensuring that populations with the greatest need—including those in socioeconomically disadvantaged rural areas—benefit more equitably.While we acknowledge that the policy's aim is to not only reduce inequities in access, but to improve the financial viability of rural practices, a future iteration, or extension of this policy, could be to consider tying incentives to patient rather than practice location. This approach has been considered in other countries. For example, in China eligibility for certain healthcare subsidies is based on patients' region of birth (Wang et al. 2014). As GPs in Australia are not restricted by catchment areas when providing telehealth services (Department of Health and Aged Care 2025b), incentives targeted to patients could potentially help increase telehealth‐based primary care in settings with under‐supply. Moreover, currently a certain number of face‐to‐face consultations with the same GP or practice within a specified period is required to enable telehealth appointments. This policy could be relaxed to increase access to telehealth for those in rural and remote areas. Alternatively, other financial incentives at the patient level could be considered to complement this model of care, such as reimbursing travel costs.

Finally, our results also highlight important institutional aspects that should be considered to optimize future policy effectiveness. We observe that out‐of‐pocket costs have been rising during this period and that the policy itself was not enough to reverse this trend. The cost of primary care is a well‐recognized issue in Australia. As aforementioned, in November 2023, the Australian Government introduced a triple bulk billing incentive for concessional cardholders and children. The current government is also proposing to expand the triple bonus to all Australians starting from November 2025 as well as a practice incentive program to pay clinics more if they bulk bill all of their patients (Department of Health and Aged Care 2023b, 2025a). Should these be implemented, future analyses should consider studying the effects on affordability and utilization of primary care. Further research into the downstream health outcomes and potential welfare implications is also warranted.

## Ethics Statement

This study was approved by the University of Melbourne Human Research Ethics Committee (12519).

## Consent

The authors have nothing to report.

## Conflicts of Interest

The authors declare no conflicts of interest.

## Permission to Reproduce Material From Other Sources

The authors have nothing to report.

## Supporting information

Supporting Information S1

## Data Availability

Data is available upon application to the Australian Bureau of Statistics.

## Endnotes

^1^
SA3 regions represent clusters of related suburbs in larger cities and regional towns, and their larger surrounding areas in less densely populated parts of Australia (AIHW 2024).

^2^
From HILDA, we sourced information on individuals' SA3 location over time. At the time of writing, we had access to 22 years of panel data; years 20.1–2022. We specifically look at migration in the years of 2021 and 2022, to best align with our analysis period.

^3^
All costs were inflated to 2022 AUD using Consumer Price Index. One USD = 1.37 AUD in 2022 (OECD 2024). Medicare Benefits Schedule (MBS) item numbers for GP visits are 3, 4, 23, 24, 36, 37, 44, 47 and benefit paid items for bulk billing management are 10,990, 10,991, 10,992, 75,855, 75,856, 75,857, 75,858 (i.e., specified as per MBS subgroup M01 “Management of bulk billed services”).

^4^
One notable exception is that the rural‐urban differences in out‐of‐pocket costs slightly increases in quarter three 2021 (by $0.43 per GP visit). As a robustness check we therefore repeat the model estimation when excluding this period. We find that when we exclude this quarter, the relative impact of the reform on our outcomes is not significantly different to our main estimation result.

^5^
Figures presented in Supporting Information S1: Appendix A.2 represent the distribution of outcomes per person per quarter. The high proportion of the sample having zero GP visits in our data in part reflects quarterly utilization patterns; that is, it is possible for a large proportion of the population to have no GP visits in a given quarter but still see a GP at some point during the year. This reflects why we also “mechanically” observe a larger concentration of zero GP visit use in the post reform data, as we have more quarters from “quarter one” which is a lower use quarter.

^6^
Area‐level socioeconomic resources are based on the 2021 SA3‐level Socioeconomic Indexes for Areas (SEIFA), with areas with higher Index of Relative Socioeconomic Deprivation (IRSD) indicating regions with relatively higher area‐level resources (Australian Bureau of Statistics 2021).
