# Supplementary material for: Do Rurality‐Based Financial Incentives Improve Equity of Primary Healthcare Access? Evidence From Australia
Source: Health Econ. 2025 Jun 18;34(9):1679–90. doi: 10.1002/hec.70000 (PMC12316583; doi:10.1002/hec.70000)
Supplement: Supplementary file 1 — Supporting Information S1 [file HEC-34-1679-s001.docx]

# Appendix

### Testing for parallel pre-trends

#### Table A. 1 – Testing for pre-reform differences in outcomes by rurality

|  | **GP visits** | **Bulk billed  GP visits** | **Out-of-pocket costs GP visits** | **Out-of-pocket costs per GP visit** |
| --- | --- | --- | --- | --- |
| Rural compared to urban – quarter 1 2021 (ref) | -0.0766^***^ [-0.085;-0.068] | -0.0946^***^ [-0.102;-0.087] | 0.720^***^ [0.564;0.876] | 1.917^***^ [1.756;2.077] |
| Rural compared to urban – quarter 2 2021 | -0.00378 [-0.016,0.008] | -0.000389 [-0.0114,0.0106] | 0.00957 [-0.211,0.230] | -0.182 [-0.404,0.0394] |
| Rural compared to urban – quarter 3 2021 | 0.013^*^ [0.001;0.025] | 0.003 [-0.008;0.014] | 0.708^***^ [0.488;0.929] | 0.428^***^ [0.203;0.652] |
| Rural compared to urban – quarter 4 2021 | -0.003 [-0.015;0.008] | 0.003 [-0.008;0.014] | 0.116 [-0.105;0.336] | 0.155 [-0.072;0.382] |

Notes: Controlling for quarter-year fixed effects, these models test whether differences between rural and urban regions as measured in quarters 2, 3, and 4 in 2021 are statistically different to the differences between rural and urban regions in quarter 1 2021.

### Distributions of outcome variables

*Figure A. 1 – GP visits*

Notes: Pre reform is quarter one 2021 to quarter four 2021. Post reform is quarter 1 2022 to quarter 3 2023.

*Figure A. 2 – Bulk billed GP visits*

Notes: Pre reform is quarter one 2021 to quarter four 2021. Post reform is quarter 1 2022 to quarter 3 2023.

*Figure A. 3 – Out of pocket cost GP visits*

Notes: Pre reform is quarter one 2021 to quarter four 2021. Post reform is quarter 1 2022 to quarter 3 2023.

*Figure A. 4 – Out of pocket cost per GP visit*

Notes: Pre reform is quarter one 2021 to quarter four 2021. Post reform is quarter 1 2022 to quarter 3 2023.

### Alternative specifications

#### **GP visits**

|  | **Poisson** | **Zero-inflated Poisson** | **Negative binomial** |
| --- | --- | --- | --- |
| Urban (Ref) | - |  | - |
| Rural | **0.898***** [0.893,0.903] | **0.916***** [0.911,0.922] | **0.888***** [0.883,0.893] |
| Post x rural | **1.022***** [1.017,1.027] | **1.018***** [1.013,1.023] | **1.025***** [1.020,1.030] |

Notes: Results are presented in exponentiated form. (1) Poisson (2) zero-inflated Poisson inflating sex and age group (3) Negative binomial. All models control for age, sex (‘male,’ ‘female’), quarter-year fixed effects. Urban regions are classified as MMM1-MMM2 and rural regions are classified as MMM3-MMM7. ^*^ *p* < 0.05, ^**^ *p* < 0.01, ^***^ *p* < 0.001

#### **Bulk billed GP visits**

|  | **Poisson** | **Zero-inflated Poisson** | **Negative binomial** |
| --- | --- | --- | --- |
| Urban (Ref) | - |  | - |
| Rural | **0.855***** [0.849,0.861] | **0.892***** [0.886,0.899] | **0.833***** [0.827,0.839] |
| Post x rural | **1.080***** [1.074,1.087] | **1.045***** [1.038,1.052] | **1.093***** [1.086,1.099] |

Notes: Results are presented in exponentiated form. (1) Poisson (2) zero-inflated Poisson inflating sex and age group (3) Negative binomial. All models control for age, sex (‘male,’ ‘female’), quarter-year fixed effects. Urban regions are classified as MMM1-MMM2 and rural regions are classified as MMM3-MMM7. ^*^ *p* < 0.05, ^**^ *p* < 0.01, ^***^ *p* < 0.001

#### **Out-of-pocket costs**

|  | **Poisson**  **OOP cost  GP visits** | **LPM Any positive OOP cost  GP visits** | **Poisson  OOP cost  GP visits conditional on positive cost** |
| --- | --- | --- | --- |
| Urban (Ref) | - |  | - |
| Rural | **1.094***** [1.082,1.105] | **0.015***** [0.014, 0.016] | **0.985***** [0.979,0.991] |
| Post x rural | **0.864***** [0.856,0.872] | **-0.027** [-0.028; -0.026] | **1.027***** [1.021,1.033] |

Notes: Poisson results are presented in exponentiated form. All models control for age, sex (‘male,’ ‘female’), quarter-year fixed effects. Urban regions are classified as MMM1-MMM2 and rural regions are classified as MMM3-MMM7. ^*^ *p* < 0.05, ^**^ *p* < 0.01, ^***^ *p* < 0.001

### Heterogeneity analyses

#### **GP visits**

#### Table A. 5 – GP visits by age group

|  | **GP visits: age group - 5-15 years** | | **GP visits: age group – 16-39 years** | | **GP visits: age group – 40-64 years** | | **GP visits: age group – 65+ years** | |
| --- | --- | --- | --- | --- | --- | --- | --- | --- |
|  | beta | 95%CI | beta | 95%CI | beta | 95%CI | beta | 95%CI |
| Rural | -0.157^***^ | [-0.165,-0.149] | -0.118^***^ | [-0.127,-0.109] | -0.0835^***^ | [-0.094,-0.073] | -0.110^***^ | [-0.129,-0.090] |
| Post x rural | 0.0395^***^ | [0.032,0.047] | 0.0387^***^ | [0.031,0.046] | 0.0173^***^ | [0.009,0.025] | 0.0231^**^ | [0.008,0.039] |
| *Percentage change relative to pre-reform rural-urban gap* | *25.2* |  | *32.8* |  | *20.7* |  | *21.0* |  |
| N | 1977481 |  | 2997269 |  | 3214068 |  | 1719344 |  |

Notes: Unit of observation is at patient-quarter level. Models control for age, sex (‘male,’ ‘female’), and quarter-year fixed effects. Rurality is based on the Monash Modified Model of remoteness with MMM1-MMM2 being classified as urban regions and MMM3-MMM7 being classified as rural regions. * p < 0.05, ** p < 0.01, *** p < 0.001.

#### Table A. 6 – GP visits by patient category

|  | **GP visits  - general** |  | **GP visits - concessional** |  |
| --- | --- | --- | --- | --- |
|  | beta | 95%CI | beta | 95%CI |
| Rural | -0.102^***^ | [-0.109,-0.0938] | -0.167^***^ | [-0.181,-0.152] |
| Post x rural | 0.0302^***^ | [0.0231,0.0373] | 0.0445^***^ | [0.0323,0.0566] |
| *Percentage change relative to pre-reform rural-urban gap* | *29.6* |  | *26.6* |  |
| N | 4250378 |  | 2626206 |  |

Notes: Unit of observation is at patient-quarter level. Models control for age, sex (‘male,’ ‘female’), and quarter-year fixed effects. Rurality is based on the Monash Modified Model of remoteness with MMM1-MMM2 being classified as urban regions and MMM3-MMM7 being classified as rural regions. * p < 0.05, ** p < 0.01, *** p < 0.001.

#### Table A. 7 – GP visits by area-level disadvantage

|  | **GP visits** | |
| --- | --- | --- |
|  | beta | 95%CI |
| Rural | -0.256^***^ | [-0.286,-0.225] |
| Rural x more disadvantaged region | 0.158^***^ | [0.127,0.189] |
| Post x rural | 0.116^***^ | [0.0883,0.144] |
| Post x rural x more disadvantaged region | -0.0941^***^ | [-0.122,-0.0659] |
| *Percentage change relative to pre-reform rural-urban gap (less disadvantage)* | *45.3* | |
| *Percentage change relative to pre-reform rural-urban gap (more disadvantage)* | *22.6* | |
| N | 1,710,104 | |

Notes: Unit of observation is at patient-quarter level. Models control for age, sex (‘male,’ ‘female’), and quarter-year fixed effects. Rurality is based on the Monash Modified Model of remoteness with MMM1-MMM2 being classified as urban regions and MMM3-MMM7 being classified as rural regions. Disadvantaged region defined as the top two quintiles ****of area-level disadvantage as per the Index Regional Socioeconomic Disadvantage (IRSD) score.**** * p < 0.05, ** p < 0.01, *** p < 0.001.

#### Table A. 9 – GP visits by rurality

|  | **GP visits – MMM3-MMM4** | | **GP visits – MMM5** | | **GP visits – MMM6-MMM7** | |
| --- | --- | --- | --- | --- | --- | --- |
|  | beta | 95%CI | beta | 95%CI |  |  |
| Rural | -0.0768^***^ | [-0.084;-0.070] | -0.112^***^ | [-0.122,-0.101] | -0.297^***^ | [-0.309,-0.285] |
| Post x rural | 0.0222^***^ | [0.016,0.028] | 0.0221^***^ | [0.0136,0.0306] | 0.0639^***^ | [0.0537,0.0742] |
| *Percentage change relative to pre-reform rural-urban gap* | *28.9* | | *19.7* | | *21.5* | |
| N | 9171217 | | 8518466 | | 8206319 | |

Notes: Unit of observation is at patient-quarter level. Models control for age, sex (‘male,’ ‘female’), and quarter-year fixed effects. Rurality is based on the Monash Modified Model of remoteness with MMM1-MMM2 being classified as urban regions and MMM3-MMM7 being classified as rural regions. * p < 0.05, ** p < 0.01, *** p < 0.001.

#### **Bulk billed GP visits**

#### Table A. 10 – Bulk billed GP visits by age group

|  | **Bulk billed GP visits: age group - 5-15 years** | | **Bulk billed GP visits: age group – 16-39 years** | | **Bulk billed GP visits: age group – 40-64 years** | | **Bulk billed GP visits: age group – 65+ years** | |
| --- | --- | --- | --- | --- | --- | --- | --- | --- |
|  | beta | 95%CI | beta | 95%CI | beta | 95%CI | beta | 95%CI |
| Rural | -0.151^***^ | [-0.159,-0.143] | -0.161^***^ | [-0.169,-0.153] | -0.109^***^ | [-0.119,-0.0994] | -0.0866^***^ | [-0.106,-0.0675] |
| Post x rural | 0.0545^***^ | [0.0476,0.0614] | 0.0980^***^ | [0.0912,0.105] | 0.0693^***^ | [0.0621,0.0764] | 0.0491^***^ | [0.0340,0.0643] |
| *Percentage change relative to pre-reform rural-urban gap* | *36.1* | | *60.9* | | *63.6* | | *56.7* | |
| N | 1977481 | | 2997269 | | 3214068 | | 1719344 | |

Notes: Unit of observation is at patient-quarter level. Models control for age, sex (‘male,’ ‘female’), and quarter-year fixed effects. Rurality is based on the Monash Modified Model of remoteness with MMM1-MMM2 being classified as urban regions and MMM3-MMM7 being classified as rural regions. * p < 0.05, ** p < 0.01, *** p < 0.001.

#### Table A. 11 – Bulk billed GP visits by patient category

|  | **Bulk billed GP visits - general** | | **Bulk billed GP visits - concessional** | |
| --- | --- | --- | --- | --- |
|  | beta | 95%CI | beta | 95%CI |
| Rural | -0.157^***^ | [-0.165,-0.150] | -0.169^***^ | [-0.183,-0.154] |
| Post x rural | 0.0913^***^ | [0.0852,0.0974] | 0.0737^***^ | [0.0618,0.0855] |
| *Percentage change relative to pre-reform rural-urban gap* | *58.1* |  | *43.6* |  |
| N | 4250378 |  | 2626206 |  |

Notes: Unit of observation is at patient-quarter level. Models control for age, sex (‘male,’ ‘female’), and quarter-year fixed effects. Rurality is based on the Monash Modified Model of remoteness with MMM1-MMM2 being classified as urban regions and MMM3-MMM7 being classified as rural regions. * p < 0.05, ** p < 0.01, *** p < 0.001.

#### Table A. 12 – Bulk billed GP visits by area-level disadvantage

|  | **Bulk billed GP visits** | |
| --- | --- | --- |
|  | beta | 95%CI |
| Rural | -0.330^***^ | [-0.355,-0.306] |
| Rural x disadvantaged region | 0.236^***^ | [0.210,0.261] |
| Post x rural | 0.181^***^ | [0.158,0.204] |
| Post x rural x disadvantaged region | -0.119^***^ | [-0.142,-0.0962] |
| *Percentage change relative to pre-reform rural-urban gap (less disadvantage)* | *54.8* | |
| *Percentage change relative to pre-reform rural-urban gap (more disadvantage)* | *66.0* | |
| N | 7,922,574 | |

Notes: Unit of observation is at patient-quarter level. Models control for age, sex (‘male,’ ‘female’), and quarter-year fixed effects. Rurality is based on the Monash Modified Model of remoteness with MMM1-MMM2 being classified as urban regions and MMM3-MMM7 being classified as rural regions. Disadvantaged region defined as the top two quintiles ****of area-level disadvantage as per the Index Regional Socioeconomic Disadvantage (IRSD) score.**** * p < 0.05, ** p < 0.01, *** p < 0.001.

#### Table A. 14 – Bulk billed GP visits by rurality

|  | **Bulk billed GP visits – MMM3-MMM4** | | **Bulk billed GP visits – MMM5** | | **Bulk billed GP visits – MMM6-MMM7** | |
| --- | --- | --- | --- | --- | --- | --- |
|  | beta | 95%CI | beta | 95%CI |  |  |
| Rural | -0.109^***^ | [-0.116,-0.102] | -0.101^***^ | [-0.111,-0.0904] | -0.281^***^ | [-0.292,-0.270] |
| Post x rural | 0.0602^***^ | [0.0547,0.0657] | 0.0645^***^ | [0.0564,0.0725] | 0.128^***^ | [0.119,0.137] |
| *Percentage change relative to pre-reform rural-urban gap* | *55.2* | | *63.9* | | *45.6* | |
| N | 9171217 | | 8518466 | | 8206319 | |

Notes: Unit of observation is at patient-quarter level. Models control for age, sex (‘male,’ ‘female’), and quarter-year fixed effects. Rurality is based on the Monash Modified Model of remoteness with MMM1-MMM2 being classified as urban regions and MMM3-MMM7 being classified as rural regions. * p < 0.05, ** p < 0.01, *** p < 0.001.

#### **Out-of-pocket costs towards GP visits**

#### Table A. 15 – Out-of-pocket cost for GP visits by age group

|  | **Out-of-pocket cost for GP visits: age group - 5-15 years** | | **Out-of-pocket cost for GP visits: age group – 16-39 years** | | **Out-of-pocket cost for GP visits: age group – 40-64 years** | | **Out-of-pocket cost for GP visits: age group – 65+ years** | |
| --- | --- | --- | --- | --- | --- | --- | --- | --- |
|  | beta | 95%CI | beta | 95%CI | beta | 95%CI | beta | 95%CI |
| Rural | -0.132^**^ | [-0.228,-0.0368] | 2.174^***^ | [1.976,2.373] | 1.079^***^ | [0.863,1.296] | -0.633^***^ | [-0.878,-0.388] |
| Post x rural | -0.594^***^ | [-0.697,-0.491] | -2.165^***^ | [-2.357,-1.973] | -1.819^***^ | [-2.013,-1.624] | -0.775^***^ | [-0.978,-0.571] |
| *Percentage change relative to pre-reform rural-urban gap* | *450.0* |  | *99.6* |  | *168.0* |  | *122.4* |  |
| N | 1977481 |  | 2997269 |  | 3214068 |  | 1719344 |  |

Notes: Unit of observation is at patient-quarter level. Models control for age, sex (‘male,’ ‘female’), and quarter-year fixed effects. Rurality is based on the Monash Modified Model of remoteness with MMM1-MMM2 being classified as urban regions and MMM3-MMM7 being classified as rural regions. * p < 0.05, ** p < 0.01, *** p < 0.001.

#### Table A. 16 – Out-of-pocket cost for GP visits by patient category

|  | **Out-of-pocket cost for GP visits - general** | | **Out-of-pocket cost for GP visits - concessional** | |
| --- | --- | --- | --- | --- |
|  | beta | 95%CI | beta | 95%CI |
| Rural | 2.745^***^ | [2.538,2.953] | 0.418^***^ | [0.259,0.576] |
| Post x rural | -2.285^***^ | [-2.479,-2.091] | -0.851^***^ | [-0.997,-0.706] |
| *Percentage change relative to pre-reform rural-urban gap* | *83.3* | | *204* | |
| N | 4250378 | | 2626206 | |

Notes: Unit of observation is at patient-quarter level. Models control for age, sex (‘male,’ ‘female’), and quarter-year fixed effects. Rurality is based on the Monash Modified Model of remoteness with MMM1-MMM2 being classified as urban regions and MMM3-MMM7 being classified as rural regions. * p < 0.05, ** p < 0.01, *** p < 0.001.

#### Table A. 17 – Out-of-pocket cost for GP visits by area-level disadvantage

|  | **Out-of-pocket cost for GP visits** | |
| --- | --- | --- |
|  | beta | 95%CI |
| Rural | 5.233^***^ | [4.308;6.158] |
| Rural x disadvantaged region | -5.300^***^ | [-6.229;-4.371] |
| Post x rural | -3.175^***^ | [-4.029;-2.322] |
| Post x rural x disadvantaged region | 1.760^***^ | [0.904;2.617] |
| *Percentage change relative to pre-reform rural-urban gap (less disadvantage)* | *60.7* | |
| *Percentage change relative to pre-reform rural-urban gap (more disadvantage)* | *N/A (no pre-reform gap)* | |
| N | 7922574 | |

Notes: Unit of observation is at patient-quarter level. Models control for age, sex (‘male,’ ‘female’), and quarter-year fixed effects. Rurality is based on the Monash Modified Model of remoteness with MMM1-MMM2 being classified as urban regions and MMM3-MMM7 being classified as rural regions. Disadvantaged region defined as the top two quintiles ****of area-level disadvantage as per the Index Regional Socioeconomic Disadvantage (IRSD) score.**** * p < 0.05, ** p < 0.01, *** p < 0.001.

#### Table A. 19 – Out-of-pocket cost for GP visits by rurality

|  | **Out-of-pocket cost for GP visits – MMM3-MMM4** | | **Out-of-pocket cost for GP visits – MMM5** | | **Out-of-pocket cost for GP visits – MMM6-MMM7** | |
| --- | --- | --- | --- | --- | --- | --- |
|  | beta | 95%CI | beta | 95%CI |  |  |
| Rural | 1.571^***^ | [1.440,1.702] | -0.542^***^ | [-0.714,-0.370] | 0.192 | [-0.0747,0.459] |
| Post x rural | -1.370^***^ | [-1.489,-1.250] | -1.403^***^ | [-1.559,-1.248] | -2.507^***^ | [-2.752,-2.262] |
| *Percentage change relative to pre-reform rural-urban gap* | *87.2* |  | *N/A (no pre-reform gap)* |  | *N/A(no pre-reform gap)* |  |
| N | 9171217 |  | 8518466 |  | 8206319 |  |

Notes: Unit of observation is at patient-quarter level. Models control for age, sex (‘male,’ ‘female’), and quarter-year fixed effects. Rurality is based on the Monash Modified Model of remoteness with MMM1-MMM2 being classified as urban regions and MMM3-MMM7 being classified as rural regions. * p < 0.05, ** p < 0.01, *** p < 0.001.

### Total number of FTE GPs by rurality 2021-2023

|  | **No. FTE GPs per 100,000 population per km^2^** | | | **No. FTE GPs per 100,000 population per km^2^ (% change)** | | |
| --- | --- | --- | --- | --- | --- | --- |
| **Year** | **MMM1-MMM2** (Urban) | **MMM3-MMM5** (Rural) | **MMM6-MMM7** (Remote) | **MMM1-MMM2**  *(Urban)* | **MMM3-MMM5**  *(Rural)* | **MMM6-MMM7**  *(Remote)* |
| 2021 | 0.02 | 2.01E-04 | 1.15E-05 | - | - | - |
| 2022 | 0.02 | 1.94E-04 | 9.50E-06 | 1.3% | -3.2% | -17.4% |
| 2023 | 0.02 | 1.93E-04 | 1.04E-05 | -1.4% | -3.9% | -9.6% |

Notes: These are averaged across SA3 regions using information on FTE GPs per SA3-year-km^2^ and the population in each SA3-year, with each SA3 being assigned the weighted average rurality measure from the Monash Modified Model (MMM) (Department of Health and Aged Care, 2023b). Information on the number of FTE GPs per SA3-year was sourced from the Department of Health and Aged Care Health Workforce Data Tool (Department of Health and Aged Care, 2023a) and the SA3-year population estimates were sourced from the Australian Bureau of Statistics (ABS, 2024).

### Weighted average working hours by rurality 2021-2023

|  | **Working hours GPs** | | | **Working hours (% change)** | | |
| --- | --- | --- | --- | --- | --- | --- |
| **Year** | **MMM1-MMM2**  *(Urban)* | **MMM3-MMM5**  *(Rural)* | **MMM6-MMM7**  *(Remote)* | **MMM1-MMM2**  *(Urban)* | **MMM3-MMM5**  *(Rural)* | **MMM6-MMM7**  *(Remote)* |
| 2021 | 42.0 | 42.5 | 45.8 | - | - | - |
| 2022 | 42.1 | 42.1 | 46.0 | 0.2% | -0.9% | 0.5% |
| 2023 | 42.0 | 42.0 | 45.2 | -0.2% | -1.2% | -1.2% |

Notes: Information on GPs’ working hours across Monash Modified Model (MMM) regions is sourced from the Department of Health and Aged Care Health Workforce Data Tool (Department of Health and Aged Care, 2023a). For each MMM region, working hours are provided in an aggregated format listing the number of GPs operating at the following capacity “1-19 hours”, “20-34 hours,” “35-49 hours,” “50-64 hours,” “65-79 hours,” and “80-125 hours.” To estimate the number of average working hours per year per MMM group, we take the midpoint of each of these categories and generate the weighted average.

# Appendix References

ABS 2024. Regional population. *In:* AUSTRALIAN BUREAU OF STATISTICS (ed.). Australian Government.

DEPARTMENT OF HEALTH AND AGED CARE 2023a. Health Workforce Data Tool. Australian Government,.

DEPARTMENT OF HEALTH AND AGED CARE 2023b. Modified Monash Model. Commonwealth of Australia.
